# Spectrochemical analysis in blood plasma combined with subsequent chemometrics for fibromyalgia detection

**DOI:** 10.1038/s41598-020-68781-x

**Published:** 2020-07-16

**Authors:** João Octávio Sales Passos, Marcelo Victor dos Santos Alves, Camilo L. M. Morais, Francis L. Martin, Antônio Felipe Cavalcante, Telma Maria Araújo Moura Lemos, Shayanne Moura, Daniel L. D. Freitas, João Vitor Medeiros Mariz, Jean Lucas Carvalho, Kássio M. G. Lima, Rodrigo Pegado

**Affiliations:** 1 Postgraduation Program in Rehabilitation Sciences, Faculty of Health Science of Trairí, Federal University of Rio Grande do Norte, Trairí St., Santa Cruz, RN 59200-000 Brazil; 2 Institute of Chemistry, Biological Chemistry and Chemometrics, Federal University of Rio Grande do Norte, Natal, 59072-970 Brazil; 3 School of Pharmacy and Biomedical Sciences, University of Central Lancashire, Preston, PR1 2HE UK; 4 Biocel Ltd, Hull, HU10 7TS UK; 5 Department of Clinical and Toxicological Analyses, Federal University of Rio Grande do Norte, Natal, Brazil

**Keywords:** Biochemistry, Fibromyalgia

## Abstract

Fibromyalgia is a rheumatologic condition characterized by multiple and chronic body pain, and other typical symptoms such as intense fatigue, anxiety and depression. It is a very complex disease where treatment is often made by non-medicated alternatives in order to alleviate symptoms and improve the patient’s quality of life. Herein, we propose a method to detect patients with fibromyalgia (*n* = 252, 126 controls and 126 patients with fibromyalgia) through the analysis of their blood plasma using attenuated total reflection Fourier-transform infrared (ATR-FTIR) spectroscopy in conjunction with chemometric techniques, hence, providing a low-cost, fast and accurate diagnostic approach. Different chemometric algorithms were tested to classify the spectral data; genetic algorithm with linear discriminant analysis (GA-LDA) achieved the best diagnostic results with a sensitivity of 89.5% in an external test set. The GA-LDA model identified 24 spectral wavenumbers responsible for class separation; amongst these, the Amide II (1,545 cm^−1^) and proteins (1,425 cm^−1^) were identified to be discriminant features. These results reinforce the potential of ATR-FTIR spectroscopy with multivariate analysis as a new tool to screen and detect patients with fibromyalgia in a fast, low-cost, non-destructive and minimally invasive fashion.

## Introduction

Fibromyalgia is a rheumatologic disorder characterized by non-articular diffuse muscle aches associated to allodynia and hyperalgesia^1^. In addition to the chronic pain, other conditions such as depression, anxiety and alterations of sleep and memory are also symptoms often found in patients with fibromyalgia^2^. These numerous symptoms of functional and emotional origin generally cause a decline in the functional, labour and social ability of patients making the treatment harder and challenging^2^. Fibromyalgia is a common disorder found in the daily clinical routine of rheumatologists and rehabilitation clinics, having an estimated incidence in 1.1 to 6.4% of the general population^1,3^. The complexity of fibromyalgia starts on its diagnosis, where the medical doctor needs to have a correct clinical interpretation of the symptoms excluding other rheumatics and neurologic diseases^2^. Gendelman et al*.*^2^ evaluated fibromyalgia diagnosis time in the primary care and observed that the disease continues having an elusive and complex diagnosis, taking even years to be properly elucidated. Patients take an average of 2.3 years and 3.7 visits to a clinical doctor in order to have a definitive diagnostic of fibromyalgia, which causes extra suffering to the patients and their families, additional medial costs and even resulting in incorrect treatments during this period^3^.

The diagnosis of fibromyalgia can be established by the classification criterion of the American College of Rheumatology (ACR) of 1990 (with the evaluation of tender points) or without the test of tender points by using a modified diagnostic criterion from ACR of 2010 or 2011^4^. In 2010, the ACR published a revised criterion set that does not count the tender points^5^. These criteria include 19 pain sites and 41 somatic symptoms^6^. In 2011, these criteria were modified by removing the 41 somatic symptoms observed by the clinical doctor and replacing them by 6 self-reported symptoms (impaired sleep, fatigue, poor cognition, headaches, depression, and abdominal pain)^6^. In Japan, these criteria presented a sensitivity and specificity to diagnostic fibromyalgia of 64% and 96%, respectively^6^; while in Germany, these criteria presented a sensitivity and specificity of 76% and 82%, respectively^7^. By comparing the different definitions of 1990 and 2010/2011, fibromyalgia is diagnosed with 86% sensitivity and 90% specificity^8^.

These studies report a wide variation of sensitivity, specificity, diagnostic patterns and pain disturbs, where the only validated method to diagnose fibromyalgia is clinical examination without any additional apparatus to aid correct diagnostic^6^. Therefore, new techniques to aid fibromyalgia screening in a fast, accurate and less-invasive fashion are needed. Novel spectrochemical analytical approaches play an important role as a new innovative technique towards clinical diagnostic^9,10^. These methods feature the use of vibrational spectroscopy techniques to analyse biological materials. Most molecules formed by covalent bonds absorb infrared (IR) radiation; amongst these, there are organic compounds containing important features of biological interest. Attenuated total reflection Fourier-transform IR (ATR-FTIR) spectroscopy enables quick and non-destructive analysis of tissue, cells or biofluids^9,11^; where, for example, a very small volume (i.e., microliters) of the latter can be used for measurement^9^. FTIR spectroscopy has been used to diagnose several types of cancer^12^, viruses^13^, neurodegenerative diseases^14^, among other conditions^15^. Fibromyalgia has been successfully differentiated from osteoarthritis and rheumatoid arthritis using FT-IR spectroscopy based on bloodspot tests^16^. Hackshaw et al*.*^16^ have detected a metabolomic fingerprint profile for fibromyalgia and other rheumatologic disorders using IR and Raman spectroscopy, where the spectrochemical signature included: CH bending in collagen, phospholipids and tryptophan; Amide III; CH in-plane bending in aromatic compounds; CH deformation, *β*-linkage and skeletal C–O–C linkage stretching for glycosaminoglycans; C–C stretching in tyrosine; and C–C twisting in phenylalanine. Moreover, studies using near-infrared spectroscopy have demonstrated great potential for fibromyalgia diagnosis using spectroscopy methods^17,18^.

The complexity of spectrochemical data requires the use of chemometric techniques in order to derive meaningful information from the sample being analysed^10^. Screening and diagnostic applications make use of multivariate classification techniques in order to distinguish and predict sample types based on their spectrochemical profile even in presence of unknown sources of variation or subtle spectral differences. Feature selection coupled with discriminant analysis techniques can provide early detection of fibromyalgia based on the sample spectrochemical profile in a quick, simple and low-cost fashion, hence, improving the disease diagnosis and patient treatment.

## Results

**Figure 1:**
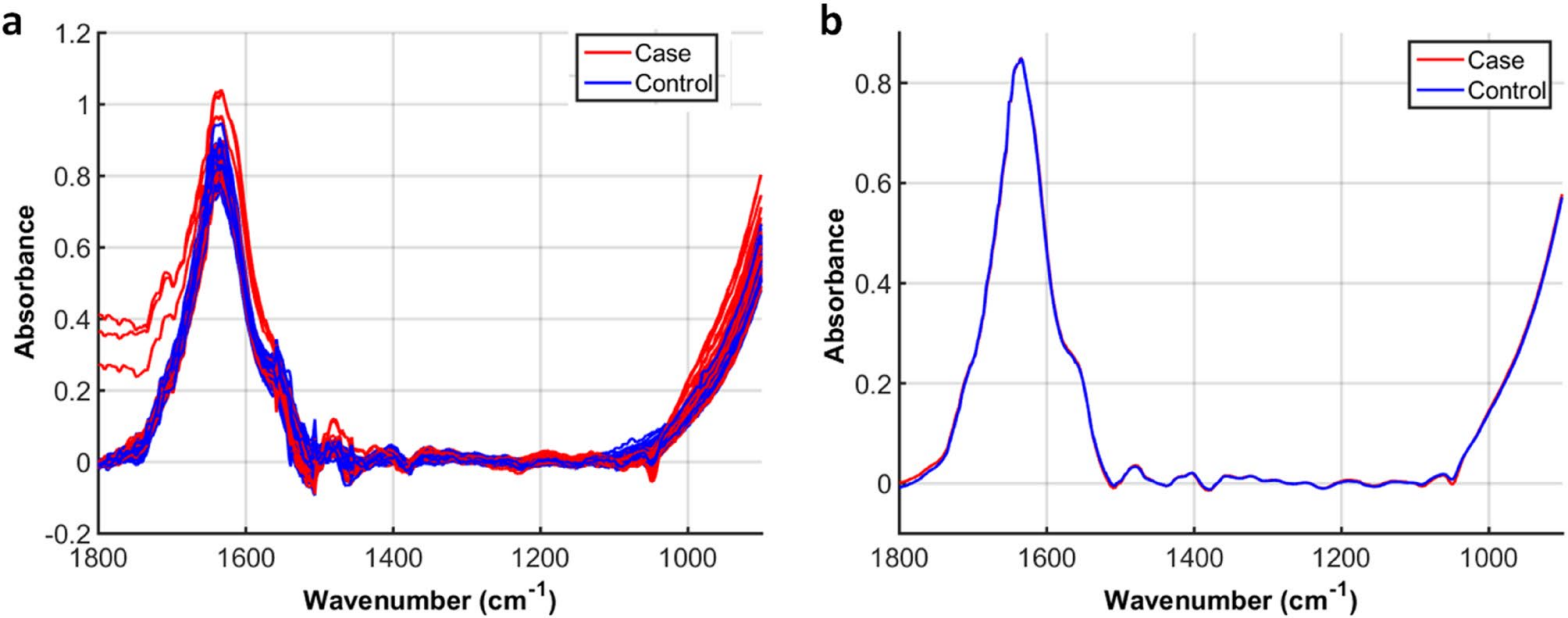
Raw and pre-processed spectra in the biofingerprint region. (**a**) Raw spectra; and (**b**) mean pre-processed spectra for case (fibromyalgia) and controls.

Socio-demographic and clinical characteristics of samples are described in Table 1. The raw spectra were pre-processed by truncating the biofingerprint region (1,800–900 cm^−1^), followed by Savitzky-Golay (SG) smoothing, automatic weighted least squares baseline correction and vector normalisation (Fig. 1). These techniques remove physical interferences from the spectra that are not associated with the analyte information, hence, highlighting the signal of interest. SG smoothing removes random noise, baseline correction minimises effects of baseline distortions common in biological materials^6^, and vector normalisation removes systematic variations associated with sample thickness or different pressures applied in the ATR module to measure the samples^10,19^. The resultant pre-processed spectra were further analysed by several chemometric techniques in order to obtain optimal results: principal component analysis with linear discriminant analysis (PCA-LDA), quadratic discriminant analysis (PCA-QDA) or support vector machines (PCA-SVM); successive projections algorithm with linear discriminant analysis (SPA-LDA), quadratic discriminant analysis (SPA-QDA) or support vector machines (SPA-SVM); and, genetic algorithm with linear discriminant analysis (GA-LDA), quadratic discriminant analysis (GA-QDA) or support vector machine (GA-SVM).

**Table 1:** Socio-demographic and clinical characteristics.

Outcomes	Fibromyalgia	Control	p value
	(Mean ± SD)	(Mean ± SD)	
Age	48.02 ± 10.03	49.84 ± 11.42	0.471
FIQ	75.03 ± 13.97	27.2 ± 21.35	0.0001
Anxiety (HAS)	38.05 ± 9.26	18.18 ± 11.93	0.001
VAS	5.74 ± 2.41	1.77 ± 2.25	0.0001
SF-36 total	53.39 ± 20.51	113.6 ± 44.58	0.0001
SF-36 physical	23.58 ± 9.17	58.69 ± 20.94	0.0001
SF-36 mental	29.79 ± 12.39	59.57 ± 19.40	0.0001
**Income**^**a**^** (%)**			0.0003
1 minimum wage	6.7	29.4	
2 to 3 minimum wage	53.3	41.2	
4 minimum wage or more	33.3	11.8	
Unreported	6.7	17.6	
**Marital status (%)**			0.03
Married	60	41.2	
Never married	26.7	41.2	
Widowed	6.7	5.9	
Divorced	6.7	11.8	
Not respond			
**Education (%)**			0.802
Elementary (incomplete)	0	5.9	
Elementary	26.7	23.5	
Secondary	26.7	41.2	
University	46.7	29.4	

Numeric data were calculated using unpaired t test. Categorical data were calculated using Chi-Square test.

*SD* standard deviation, *FIQ* Fibromyalgia Impact Questionnaire, *VAS* Visual Analogue Scale; *HAS* Hamilton Anxiety Scale, *SF-36* Short Form 36 Health Survey.

^a^Brazilian National Minimum Wage, US$ 252.14 per month.

Before model construction, 70% of samples were assigned to the training set, 15% to the validation set, and 15% to the test set using the Kennard-Stone uniform sampling algorithm^20^. The training set was used for model construction, the validation set for internal model optimisation, and the test set for final model evaluation, where figures of merit (accuracy, sensitivity and specificity) reflecting the model performance towards external samples were calculated. The accuracy represents the total number of samples correctly classified considering true and false negatives; the sensitivity represents the proportion of positives that are correctly classified; and the specificity represents the proportion of negatives that are correctly classified^21^.

**Figure 2:**
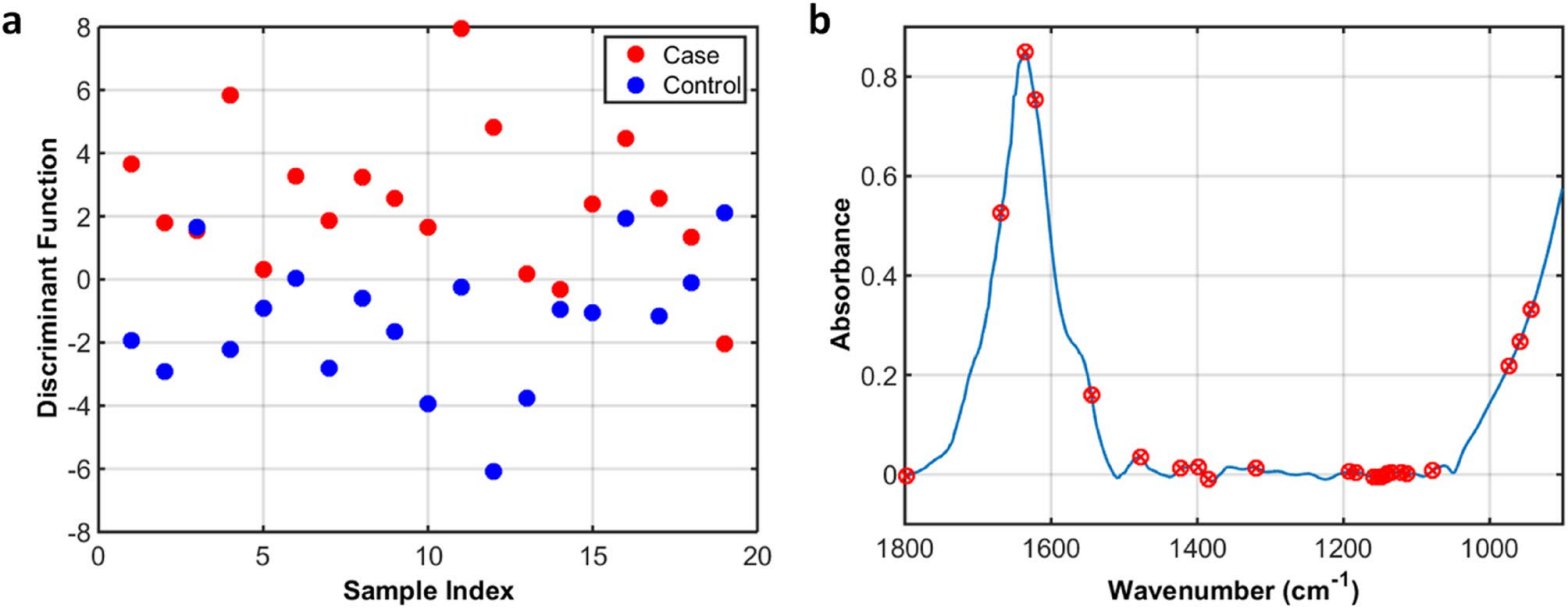
GA-LDA results for classifying case (fibromyalgia) and controls. (**a**) Discriminant function graph for the samples in the test; and (**b**) GA-LDA selected wavenumbers.

Table 2 illustrates the model performance of the algorithms tested. The best results were obtained by GA-LDA, with 84.2% accuracy, 89.5% sensitivity and 79.0% specificity. These metrics demonstrate a satisfactory classification rate for distinguishing the two groups (case vs. control). The model potential for class separation in the test set can be observed in Fig. 2a, where the data dispersion in terms of the discriminant function score is shown.

**Table 2:** Figures of merit for different algorithms applied to classify case (fibromyalgia) and controls in the test set.

Algorithm	Accuracy (%)	Sensitivity (%)	Specificity (%)
PCA-LDA	60.5	68.4	52.6
PCA-QDA	65.8	73.7	57.9
PCA-SVM	68.4	78.9	57.9
SPA-LDA	63.1	57.9	68.4
SPA-QDA	63.2	68.4	57.9
SPA-SVM	70.1	73.7	68.4
GA-LDA	**84.2**	**89.5**	**79.0**
GA-QDA	60.5	47.4	73.7
GA-SVM	57.9	57.9	57.9

The best algorithm (GA-LDA) is highlighted in bold.

The selected wavenumbers by GA-LDA are shown in Fig. 2b. These wavenumbers responsible for class separation were: 943; 959; 974; 1,078; 1,113; 1,121; 1,134; 1,140; 1,142; 1,148; 1,153; 1,159; 1,182; 1,192; 1,319; 1,385; 1,398; 1,423; 1,477; 1,545; 1,622; 1,636; 1,668; and, 1,798 cm^−1^. Their tentative biomarkers assignment^22^ are shown in Table 3.

**Table 3:** Tentative assignment of the spectral markers selected by GA-LDA.

Wavenumber (cm^−1^)	Tentative assignment
943	Phosphodiester region
959	Symmetric stretching vibration of *ν*_1_ (PO_4_^3−^)
974	OCH_3_ stretching in polysaccharides
1,078	Phosphate I in RNA
1,113	P-O-C symmetric stretching
1,121	Symmetric phosphodiester stretching band RNA
1,134	C–OH stretching band in oligosaccharide
1,140	C–O stretching in phosphate and oligosaccharides
1,142	C–O stretching in phosphate and oligosaccharides
1,148	C–O stretching in carbohydrates
1,153	Stretching vibrations of hydrogen-bonding C–OH groups
1,159	*ν*(C–O) of proteins and carbohydrates
1,182	Amide III
1,192	Collagen
1,319	Amide III
1,385	*δ*(CH_3_)
1,398	CH_3_ symmetric deformation
1,423	*ν*_s_(COO_2_) in polysaccharides or pectin
1,477	*δ*(CH_2_) of the methylene chains in lipids
1,545	Amide II in proteins
1,622	Peak of nucleic acids due to the base carbonyl stretching and ring breathing mode
1,636	*β*-sheet structure of Amide I
1,668	Amide I (anti-parallel *β*-sheet)
1,798	*ν*(C=C) in lipids

*ν*: stretching vibration; *δ*: bending vibration.

## Discussion

According to studies made by the American College of Rheumatology for diagnosis and classification of fibromyalgia^2^, groups of patients with fibromyalgia present a good differentiation from a control cohort containing patients with rheumatologic pains (but not fibromyalgia) with an accuracy of 84.9% and sensitivity of 88.4% using the traditional diagnostic through the combination of generalised pains analysis in 11 to 18 pain sites. These reference metrics are very similar to our approach using spectrochemical analyses coupled to chemometric techniques, where we achieved an accuracy of 84.2% and sensitivity of 89.5%. Twenty-four spectral wavenumbers were selected by GA-LDA as being responsible for case–control separation (Table 3). Among these are carbohydrate, nucleic acid, protein and lipid absorptions^9^, which are often regions of alterations for disease diagnosis.

Since the samples were measured in the liquid state, water is an interference in the spectral signal compressing and masking the absorbance of some bands, and changing the fingerprint region spectral profile in comparison with dry samples^23,24^. Water mainly affects the IR spectrum outside the fingerprint region, with strong bands at 3,300–3,400 cm^−1^ (hydrogen-bonded O–H stretching) and 3,600–3,650 cm^−1^ (free O–H stretching)^10^, which were regions removed from the spectra before analysis; however, water has an absorbance in the fingerprint region at 1646 cm^−1^ that adds to the Amide I signal of proteins^23^, hence, broadening the band and transforming the Amide II band in a small arm on the right-hand side of the Amide I absorption band. Additionally, absorbances below 1,000 cm^−1^ tend to greatly increase in liquid environment^23^, compressing the bands between ~ 1,200 and 1,500 cm^−1^_._

Performing experiments with liquid samples has some benefits, since the spectral measurement is faster and the experimental setup is simpler than analysing dry samples^24^, which provides a higher throughput capability. However, liquid samples are affected by water interference, which compromises the sensitivity and detection limit of the technique. In addition, the ATR crystal must be cleaned more rigorously between measurements in order to remove biomolecules, particularly absorbed proteins, onto the crystal surface^24^. Overall, measuring liquid samples increases the analytical frequency of the technique but it also brings high-risks, especially associated with water interference, thus one must be careful when using liquid biopsies for spectroscopy measurements. Herein, the water interference seems not to be an issue that affects sample discrimination, once a relatively high discriminant performance was obtained to distinguish the samples based on the spectral profiles; but sample type, among other factors such as type of substrate, sample volume or the way the sample is deposited^25^, must be investigated during the pre-analytical phase for other applications once water among other interferences may severely affect important spectral regions. Therefore, the sampling method (liquid or dry samples) depends on the application of interest, the analytical frequency desired, and the degree of sample discrimination being pursued. Obtaining satisfactory results is an empirical balance of these factors.

Hackshaw et al*.*^16^ have used FT-IR spectroscopy to differentiate 14 fibromyalgia patients from those with osteoarthritis (*n* = 12) and rheumatoid arthritis (*n* = 15) based on a bloodspot test. The samples were pre-treated by removing proteins with molecular weight over 10 kDa via ultrafiltration, therefore, the analysis was focused on low-weight metabolites. The spectral data were multivariately analysed using soft independent modeling class analogy (SIMCA) algorithm, where a perfect separation with zero misclassifications (100% accuracy) was obtained; on the other hand, using a metabolomic approach based on ultrahigh performance liquid chromatography/tandem mass spectrometry (UHPLC/MS/MS) and gas chromatography/mass spectrometry (GC/MS), the accuracy was substantially inferior at 75%. Hackshaw et al.^26^ also metabolically profiled fibromyalgia (*n* = 50) and other rheumatologic disorders (rheumatoid arthritis (*n* = 29), osteoarthritis (*n* = 19) and systemic lupus erythematosus (*n* = 23)) using FT-IR and Raman spectroscopy based on bloodspot samples, where the different disorders were classified with 100% accuracy using SIMCA algorithm. Apart from metabolomic changes^16,26^, Raffaeli et al*.*^27^ identified a Mu opioid receptor, expressed on the B lymphocytes surface, as a biological marker (named Mu-Lympho-Maker) for an objective chronic pain diagnosis of fibromyalgia.

The updated criteria to improve fibromyalgia detection^3^ propose clinical improvements for patient screening without the need of extensive physical tests, such as the test of pain sites, and focusing on alternative diagnostic criteria based on symptoms severity; however, it does not replace or overcome the statistical metrics found in the previous discussed study^3^. In studies involving different diagnostic criteria^2,3^, it is possible to observe improvements in the criteria from 2010 by adding different patient conditions such as cognitive dysfunction, depression and anxiety^23^. The inclusion of wider symptomatic criteria may improve the accuracy, sensitivity and specificity for fibromyalgia diagnosis; but it is still a time-consuming and somewhat subjective test. ATR-FTIR coupled to chemometrics has the potential to replace or aid fibromyalgia screening based on a small aliquot of blood plasma, thus speeding and adding an extra degree of objectiveness for fibromyalgia diagnosis.

Fibromyalgia remains a very complex and elusive diagnosis with negative impairment of economic and financial burden that include laboratory and imaging exams, frequent visits to specialists, and different courses of treatments^1^. Gendelman et al*.*^1^ suggest that specific patients characteristics including age and comorbidities could infer the time for diagnosis. Furthermore, physicians’ skill-related factors (poor communication, age and the knowledge of the ACR diagnostic criteria) contribute to diagnostic inaccuracy^1,3^. The lack of a definitive diagnosis of fibromyalgia potentially affect functionality, daily activities and work productivity^5,6^. Although the pattern of clinical diagnosis of fibromyalgia continues to improve in terms of accuracy and sensitivity, there is an increasing recognition of both misdiagnosis and overdiagnosis^1^. Spectrochemical analysis with multivariate classification techniques could provide and additional option for clinical diagnosis of fibromyalgia.

This study provides a blood-based test for fibromyalgia, where the combination of chemometric and spectrochemical methods contribute for fibromyalgia diagnosis. Efforts to improve earlier diagnosis of fibromyalgia is crucial to reduce functional deficits, costly treatments, and provide better rehabilitation protocols. These results reinforce the potential of ATR-FTIR spectroscopy with multivariate analysis as a new tool to screen and detect patients with fibromyalgia in a fast, low-cost, non-destructive and minimally invasive fashion. Additional studies comparing fibromyalgia with others chronic rheumatic diseases, such as osteoarthritis, rheumatoid arthritis and chronic fatigue syndrome, are necessary for potential diagnosis.

## Methods

### Samples

This case–control study was performed following the ethics standards of the Declaration of Helsinki and was approved by the local institutional ethics committee at the Onofre Lopes University Hospital (Federal University of Rio Grande do Norte, Natal, Brazil) under registration number 2.631.168. Informed consents were obtained from all subjects of this study; and all experimental protocols complied with the ethics guidelines. Subjects were recruited from social media and at the medical clinic of the Onofre Lopes University Hospital (HUOL). The following inclusion criteria were adopted: (a) medical diagnosis of fibromyalgia according to the ACR/2010; (b) ability to answer questionnaire and understand this study aim; (c) patients not undergoing physical therapy or rehabilitation programs during the three previous months; and (d) age ranging from 18 to 80 years old. The exclusion criteria were: (a) physical and/or organic problems, when these compromised questionnaire applications; and, (b) rheumatic and/or autoimmune diseases including chronic fatigue syndrome, rheumatoid arthritis, gout and lupus.

A total of 126 fibromyalgia patients and 126 control subjects were enrolled in this study. The data were collected from July 2018 to March 2019 and the recruitment was performed during this entire period. The study was conducted at the Clinical and Epidemiological Laboratory at the Federal University of Rio Grande do Norte, Natal, Brazil. Socio-demographic data (gender, age, education level, occupation, marital status, and ethnicity), clinical data (fibromyalgia impact, anxiety, pain, and quality of life), and 10 mL of blood were collected from each patient in the same day.

### Clinical measurements

The functional capacity was evaluated using the Brazilian version of the Fibromyalgia Impact Questionnaire (FQI), which is a self-administered questionnaire that measures the functional aspects of the patient^28^. FIQ contains three Likert-scale-type questions (levels of response) and seven visual analogue questions. All the scales vary from 1 to 10 and a high score indicates negative impact and more seven symptoms. The total FIQ score is graded from 1 to 100 points. Higher scores are related to greater impact of the disease on the patients’ functionality and a corresponding reduction in their quality of life^28^.

The severity and anxiety symptoms were measured using the Hamilton Anxiety Scale (HAS)^29^. The HAS was administered by an interviewer who asked a series of semi-structured questions related to symptoms of anxiety. The interviewer rated the individuals on a five-point scale for each of the 14 items^29^. Seven of the items specifically address psychic anxiety and the remaining seven somatic anxiety. The values on the scale range from 0 to 4: 0—there is no anxiety; 1—mild anxiety; 2—moderate anxiety; 3—severe anxiety; 4—very severe or grossly disabling anxiety. The total anxiety score ranges from 0 to 56^29^.

The visual analogue scale (VAS) was used to measure pain. VAS is a unidimensional measure of pain intensity, which has been widely used in diverse adult populations^30^. The VAS pain is a continuous scale comprised of a horizontal line, usually 10 cm (100 mm) in length, anchored by 2 verbal descriptors, one for each extreme symptom^30^. For pain intensity, the scale is most commonly anchored by “no pain” (score of 0) and “pain as bad as it could be” or “worst imaginable pain” (score of 100 [100 mm scale]). The VAS is administered, and the patient is asked to indicate the distance on the 0 to 100 mm line on the segmented scale that best describes their pain intensity in the last 24 h^30^.

Quality of life (QoL) was assessed by the Short Form 36 Health Survey (SF-36)^31^. The SF-36 is a generic tool that measures eight general health concepts: physical functioning, physical role, bodily pain, general health, vitality, social functioning, role emotional, and mental health^31^. Two main scores are available to summarise these scales: physical composite score (PCS) and mental composite score (MCS), and the total SF-36 score. All these scores fall within 0–100 scale, with higher scores reflecting better QoL^31^.

### Spectrochemical analyses

The samples were stored at − 15 °C before spectrochemical analysis. Measurements were performed at the Institute of Chemistry of the Federal University of Rio Grande do Norte, Natal, Brazil. A Bruker Vertex 70 FTIR spectrometer (Bruker, Coventry, UK) coupled to an ATR Helios attachment was used for spectral acquisition. Spectra were acquired with 32 scans (4 cm^−1^ resolution) and in triplicate for each sample. Before every new sample the ATR crystal was cleaned, and a new background was set in order to account for ambient variability. The blood plasma samples were measured in the liquid state.

### Data analysis

The spectral data were processed using the MATLAB R2014b software (MathWorks Inc., Natick, USA) with the PLS Toolbox version 7.8 (Eigenvector Research Inc., Wenatchee, USA) and lab-made routines. The spectral data were initially cropped to the bio-fingerprint region (900–1,800 cm^−1^) and pre-processed by automatic weighted least squares baseline correction and vector normalisation. The samples’ spectra were then divided into training (70%), validation (15%) and test (15%) sets using the Kennard-Stone uniform sample selection algorithm^20^. The training set is used for model construction, the validation set for model internal validation and optimization, and the test set for evaluating the model predictive performance towards external samples through the calculation of figures of merit (accuracy, sensitivity and specificity). Several algorithms of feature extraction and selection coupled to discriminant analysis techniques were tested on the spectral data; these were: principal component analysis linear discriminant analysis (PCA-LDA), principal component analysis quadratic discriminant analysis (PCA-QDA), principal component analysis support vector machines (PCA-SVM), successive projections algorithm linear discriminant analysis (SPA-LDA), successive projections algorithm quadratic discriminant analysis (SPA-QDA), successive projections algorithm support vector machines (SPA-SVM), genetic algorithm linear discriminant analysis (GA-LDA), genetic algorithm quadratic discriminant analysis (GA-QDA), and genetic algorithm support vector machines (GA-SVM).

PCA decomposes the pre-processed spectral data into a small number of principal components (PCs) that are orthogonal to each other and explain most of the original data variance. Each PC is composed of scores, representing the variance on sample direction, hence, being used to assess similarities/dissimilarities between the samples; and loadings, representing the variance on wavenumber direction, thus being used to assess variable importance. Therefore, PCA can be used for data reduction, feature extraction, pattern recognition, sample selection, exploratory analysis, among other^32^. Successive projections algorithm (SPA) and genetic algorithm (GA) are forward feature selection algorithms that select sets of wavenumbers responsible for maximizing class differences. SPA^33^ is a forward feature selection method that works by minimizing the data multicollinearity through a series of projections of the original wavenumbers in an iterative way. GA^34^ is another iterative method that works based on the principle of natural evolution where a set of wavenumbers (chromosomes) undergo an evolution-like model of combinations, cross-overs and mutations until the best set of wavenumbers achieve the best fitness according to a pre-determined cost-function that maximizes class differences.

The outputs from PCA (scores), SPA and GA can be used as input variables for discriminant analysis. LDA and QDA are discriminant analysis techniques based on a Mahalanobis distance calculation between the samples, where the LDA ($${L}_{ik})$$ and QDA ($${Q}_{ik}$$) classification scores are calculated as follows^35^:

1 $${L}_{ik}={\left({\mathbf{x}}_{i}-{\stackrel{-}{\mathbf{x}}}_{k}\right)}^{T}{\mathbf{C}}_{pooled}^{-1}\left({\mathbf{x}}_{i}-{\stackrel{-}{\mathbf{x}}}_{k}\right)-2{log}_{e}{\pi }_{k}$$

2 $${Q}_{ik}={\left({\mathbf{x}}_{i}-{\stackrel{-}{\mathbf{x}}}_{k}\right)}^{T}{\mathbf{C}}_{k}^{-1}\left({\mathbf{x}}_{i}-{\stackrel{-}{\mathbf{x}}}_{k}\right)+{log}_{e}\left|{\mathbf{C}}_{k}\right|-2{log}_{e}{\pi }_{k}$$

where ${\mathbf{x}}_{i}$ is vector containing the input variables for sample *i*; ${\stackrel{-}{\mathbf{x}}}_{k}$ is the mean vector of class *k*; ${\mathbf{C}}_{pooled}$ is the pooled covariance matrix; ${\mathbf{C}}_{k}$ is pooled variance–covariance matrix of class *k*; and ${\pi }_{k}$ is the prior probability of class *k*. The SVM classification takes the form^36^:

3 $$f\left(x\right)=sign\left(\sum_{i=1}^{{N}_{SV}}{\alpha }_{i}{y}_{i}K\left({\mathbf{x}}_{i},{\mathbf{z}}_{j}\right)+b\right)$$

$K\left({\mathbf{x}}_{i},{\mathbf{z}}_{j}\right)$ is the kernel function for ${\mathbf{x}}_{i}$ and ${\mathbf{z}}_{j}$ which are input variables for different classes; ${\alpha }_{i}$ is the Lagrange multiplier; ${y}_{i}$ is the training class membership; and $b$ is the bias parameter.
